# A ResNet-LSTM hybrid model for predicting epileptic seizures using a pretrained model with supervised contrastive learning

**DOI:** 10.1038/s41598-023-43328-y

**Published:** 2024-01-15

**Authors:** Dohyun Lee, Byunghyun Kim, Taejoon Kim, Inwhee Joe, Jongwha Chong, Kyeongyuk Min, Kiyoung Jung

**Affiliations:** 1 Department of Computer Science, Hanyang University, Seoul, 04763 South Korea; 2 Department of Neurology, Ajou University School of Medicine, Suwon, 16499 South Korea; 3 Department of Computer Science, State University of New York Korea, Incheon, 21985 South Korea; 4 Present Address: Department of Electronics Engineering, Hanyang University, Seoul, 04763 South Korea; 5 Department of Neurology, Seoul National University College of Medicine, Seoul, 03080 South Korea

**Keywords:** Neuroscience, Diseases of the nervous system, Epilepsy

## Abstract

In this paper, we propose a method for predicting epileptic seizures using a pre-trained model utilizing supervised contrastive learning and a hybrid model combining residual networks (ResNet) and long short-term memory (LSTM). The proposed training approach encompasses three key phases: pre-processing, pre-training as a pretext task, and training as a downstream task. In the pre-processing phase, the data is transformed into a spectrogram image using short time Fourier transform (STFT), which extracts both time and frequency information. This step compensates for the inherent complexity and irregularity of electroencephalography (EEG) data, which often hampers effective data analysis. During the pre-training phase, augmented data is generated from the original dataset using techniques such as band-stop filtering and temporal cutout. Subsequently, a ResNet model is pre-trained alongside a supervised contrastive loss model, learning the representation of the spectrogram image. In the training phase, a hybrid model is constructed by combining ResNet, initialized with weight values from the pre-trained model, and LSTM. This hybrid model extracts image features and time information to enhance prediction accuracy. The proposed method’s effectiveness is validated using datasets from CHB-MIT and Seoul National University Hospital (SNUH). The method’s generalization ability is confirmed through Leave-one-out cross-validation. From the experimental results measuring accuracy, sensitivity, and false positive rate (FPR), CHB-MIT was 91.90%, 89.64%, 0.058 and SNUH was 83.37%, 79.89%, and 0.131. The experimental results demonstrate that the proposed method outperforms the conventional methods.

## Introduction

Epilepsy is a chronic neurological disorder that affects about 50 million people, which is approximately 1% of the world’s population. Seizures are typical clinical manifestations of epilepsy, characterized by sudden and temporary neurobehavioral symptoms caused by abnormally hypersynchronous electrical discharges from overexcited neurons in the brain^1,2^. Except for a few special cases, seizures occur irregularly, and patient’s premonitory symptoms are uncertain. Moreover, the exact onset time cannot be estimated because it differs among individuals. Because of this unpredictability, people with epilepsy are limited in their social activities and exposed to trauma and danger, which substantially impacts their quality of life^3^. Furthermore, patients with severe epilepsy are hospitalized and managed throughout the day by medical personnel. However, the medical personnel are insufficient to manage all patients, and correct judgments cannot be made based solely on patient behavior monitoring. As a result, various studies related to epilepsy are being conducted to ensure the stability of daily lives for epilepsy patients^4^ and enable precise prevention and treatment with limited medical resources.

Due to the fact that EEG detects electric signals generated by the brain during seizures, absence seizures and focal seizures without awareness can also be identified^5^. Therefore, from the 1970s to the present, EEG data has been widely utilized in seizure prediction studies. Research fields relating to epilepsy are primarily separated into seizure detection^6–8^ and prediction. Both studies are essential, and current research mostly focuses on seizure prediction, starting with seizure detection. Early studies of seizure prediction included manual feature extraction techniques, which are unsuitable for deriving distinct patterns from massive datasets. Consequently, recent research on seizure prediction employs deep learning algorithms suitable for recognizing complicated patterns in large datasets.

Our main contributions can be summarized as follows: (1) we propose a pre-processing method in which EEG data compensates for the deficiencies of training data in deep learning and makes ResNet advantageous for feature extraction. (2) we pre-train the image representation to achieve the best performance possible with small amounts of data. (3) To extract various features from time-sequence image data, we propose a hybrid model combining ResNet and LSTM.

## Related work

The presence of differences between pre-ictal and inter-ictal brain waves was the fundamental assumption of seizure prediction^9,10^. Philippa *et al.* and Shufang Li *et al.* linearly assessed the spike rate in the segment using an EEG raw signal to predict seizures^11^. Ali Shahidi Zandi *et al.* collected features using the positive zero-crossing approach and forecasted them by classifying inter-ictal and pre-ictal seizures using the Bayesian Gaussian Mixture model^12^, respectively. Dongrae Cho*et al.* decomposed spectral components using various filtering techniques, including bandpass filtering, emergency mode degradation, and multivariate empirical mode degradation, and made predictions by comparing the phase synchronization of the gamma frequency band to that of other frequency bands^13^. The majority of past research has focused on signal analysis techniques, which are unsuitable for irregular and complex EEG data. To extract the frequency components of EEG data, numerous researchers use empirical mode decomposition^14^, continuous wavelet transform (CWT)^15^, discrete wavelet transform (DWT)^16^, and STFT^17^. Furthermore, many efforts have been made to extract meaningful information from EEG data, such as principal component analysis (PCA)^16^, approximate entropy^18^, and the Hjorth parameter^19^.Various classifiers classified the extracted features as pre-ictal and inter-ictal. Machine learning techniques such as Bayesian Gaussian Mixture^12^, Support Vector Machine (SVM)^20,21^, and K-Nearest Neighbor (KNN)^22^ have begun showing impressive outcomes. In addition, recent research employing deep learning models, which is closely related to this study, has shown advanced results. In contrast to prior studies, Haidar Khan *et al.* transformed signals using the Wavelet Transform (WT) and projected changes in the probability distribution and Convolution Neural Networks (CNN) using Kullback-Leibler divergence (KL divergence), a probability distribution of data method. Kostas *et al.* predicted, using an LSTM model capable of reflecting the information of time sequence signals^23^. Liu *et al.* proposed a novel patient-independent approach in epilepsy research by applying the advanced form of LSTM known as the Bidirectional Long Short-Term Memory (Bi-LSTM) network^24^. Until recently, research utilizing CNN-based deep learning models such as 3D-CNN^25,26^ and ResNet^27^ have been used as classification methods.

Extraction and classification methods, as described in the previous study, are equally crucial for all classification algorithms. When determining the method for feature extraction and classification, it is necessary to consider the characteristics and limitations of the data. Variable patient characteristics make it difficult to use EEG data for patient-independent seizure prediction^28^. Therefore, we performed patient-specific seizure prediction. Data scarcity is a disadvantage of patient-specific methods. Additionally, the inherent disadvantages of EEG data are their complexity and irregularity^29^. To achieve superior performance with limited data, we propose a specific pre-trained model consisting of ResNet and supervised contrastive loss. We also augment the data with a band-stop filter and temporary cutout. Moreover, we propose a hybrid model that combines ResNet and LSTM to reflect both types of information during training. We use STFT to transform the irregular and complex shortcomings of the EEG into data with frequency-time information.

## Database

The dataset used in this research can be classified according to the reference electrode selection method, using two methods: ’Unipolar reference’ and ’Bipolar reference’. The SNUH dataset was measured using the ’Unipolar reference’ method, while the CHB-MIT dataset used the ’Bipolar reference’ method. In the ’Unipolar reference’ method, the GND value is determined by averaging all the electrodes and converting them into digital signals, with all electrodes sharing the same GND. The difference between the individual signal and the signal measured at the common ground is recorded. However, this method is susceptible to fine noise or common-mode signals, which can also be amplified and output. On the other hand, in the ’Bipolar reference’ method, each adjacent electrode is used as a GND to convert it into a digital signal. This method is resistant to noise from the common signal between electrode attachment points, as the measurement procedure eliminates it. However, it makes it difficult to observe brain waves at a specific location. A description of the two datasets is included below.

### CHB-MIT scalp EEG dataset

The CHB-MIT dataset serves as a validated dataset primarily utilized in research related to seizure detection and prediction. It comprises data gathered from Children’s Hospital Boston, encompassing a total of 844 hours of data and 245 recorded seizures. The scalp EEG information was recorded using 22 electrodes with a sampling rate of 256Hz and extracted using the bipolar method. Among the 22 electrode channels, 18 common channels (’FP1-F7’, ’F7-T7’, ’T7-P7’, ’P7-O1’, ’FP1-F3’, ’F3-C3’, ’C3-P3’, ’P3-O1’, ’FP2-F4’, ’F4-C4’, ’C4-P4’, ’P4-O2’, ’FP2-F8’, ’F8-T8’, ’T8-P8’, ’P8-O2’, ’FZ-CZ’, ’CZ-PZ’) were used for training purposes.

### SNUH scalp EEG dataset

This study was approved by the Institutional Review Board of the Seoul National University Hospital (IRB No. H-1710-030-891). Written informed consent from the patients was waived by the Institutional Review Board of Seoul National University Hospital. All methods were carried out in accordance with relevant guidelines and regulations. The SNUH dataset was collected from Seoul National University Hospital and included 845 h of data and 78 seizures. Scalp EEG information was recorded using 21 electrodes with a sampling rate of 200 Hz and was extracted with a unipolar reference. All 21 electrode channels (’Fp1-AVG’, ’F3-AVG’, ’C3-AVG’, ’P3-AVG’, ’Fp2-AVG’, ’F4-AVG’, ’C4-AVG’, ’P4-AVG’, ’F7-AVG’, ’T1-AVG’, ’T3-AVG’, ’T5-AVG’, ’O1-AVG’, ’F8-AVG’, ’T2-AVG’, ’T4-AVG’, ’T6-AVG’, ’O2-AVG’, ’Fz-AVG’, ’Cz-AVG’, ’Pz-AVG’) were used for training.

**Figure 1:**
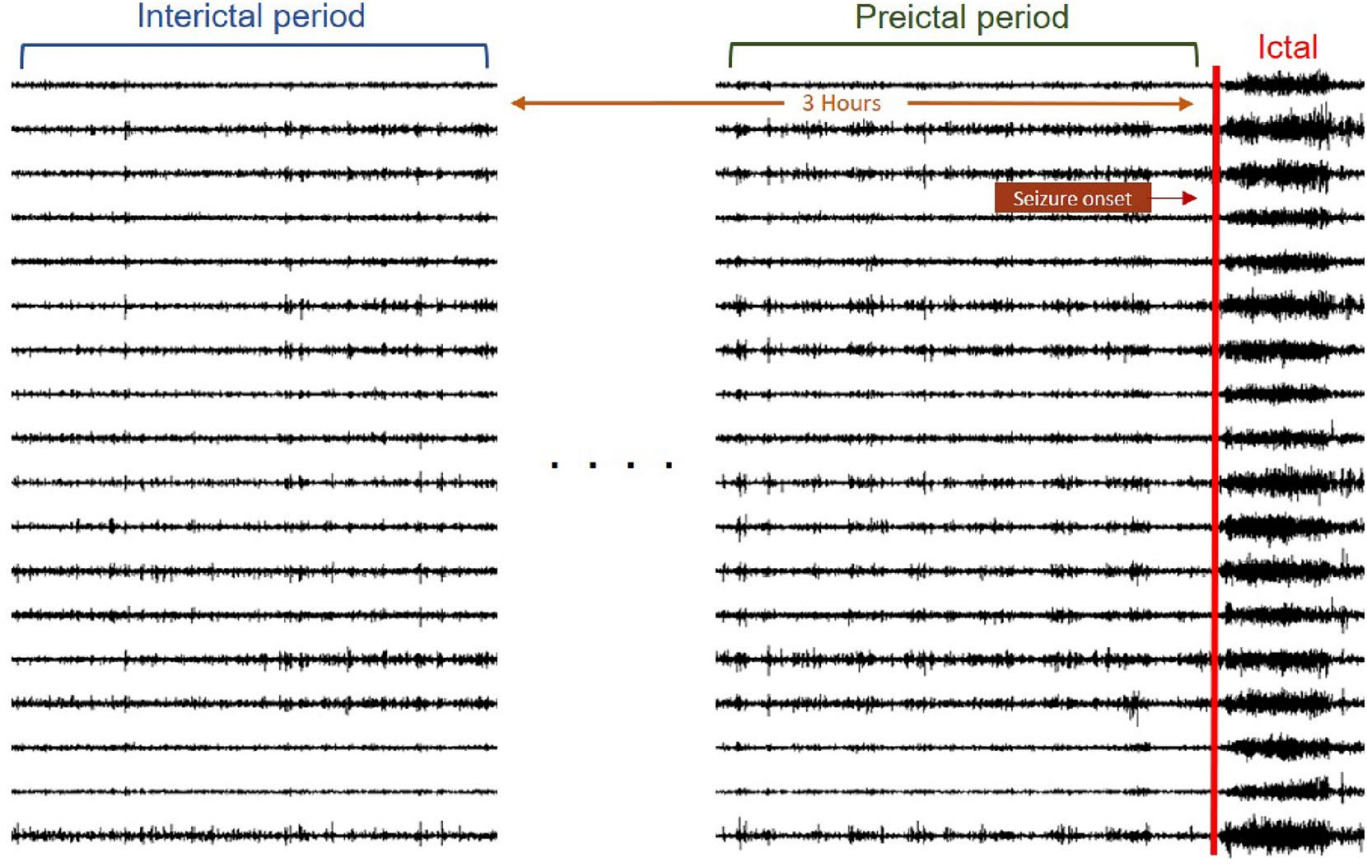
Definition of pre-ictal and inter-ictal period.

## Pre-processing

The amount of data, the model, and the characteristics of the data all significantly influence the performance of models in data-based supervised learning. EEG data has three disadvantages: a class imbalance between pre-ictal and inter-ictal, an insufficient data quantity, and the complexity and irregularity of the data, making analysis difficult. These disadvantages directly affect the model’s performance. We addressed these issues during the pre-processing phase.

### Data sampling

**Figure 2:**
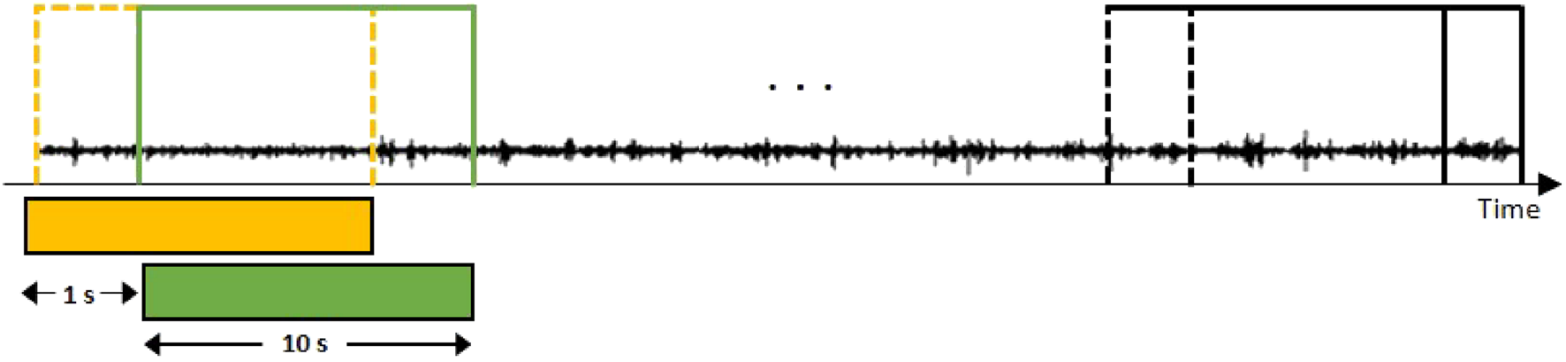
The sliding window algorithm is employed to apply oversampling with a 10 s window that overlaps by 1 s.

As illustrated in Fig. 1, we defined the period before ictal onset as “pre-ictal” and set the durations to 10, 15, and 30 min. “Inter-ictal” is defined as the period more than 3 hours away from the seizure, when the seizure waveform is absent from the EEG^30^. The validation datasets, CHB-MIT and SNUH, exhibit a class imbalance between pre-ictal and inter-ictal due to the relatively small number of ictals in comparison to the total length. When there is a large difference in the number of classes in the dataset, classes with a high distribution are given more weight during training. In the case of a seizure dataset with a substantial proportion of inter-ictal, overall accuracy may increase while sensitivity decreases. As sensitivity directly related to the patient’s life, resolving the difference in distribution between the two classes can lead to improved performance. To resolve the imbalance, we employed undersampling to extract data of the same length as pre-ictal and inter-ictal data, as depicted in Fig. 1. Additionally, oversampling was conducted to supplement the existing limited data and compensate for information loss during undersampling. As shown in Fig. 2, the window size was set to 10 s, and the sliding window algorithm was applied every 1s to generate overlapping data. Through data sampling, the data imbalance was resolved, and insufficient data were supplemented.

### STFT

**Figure 3:**
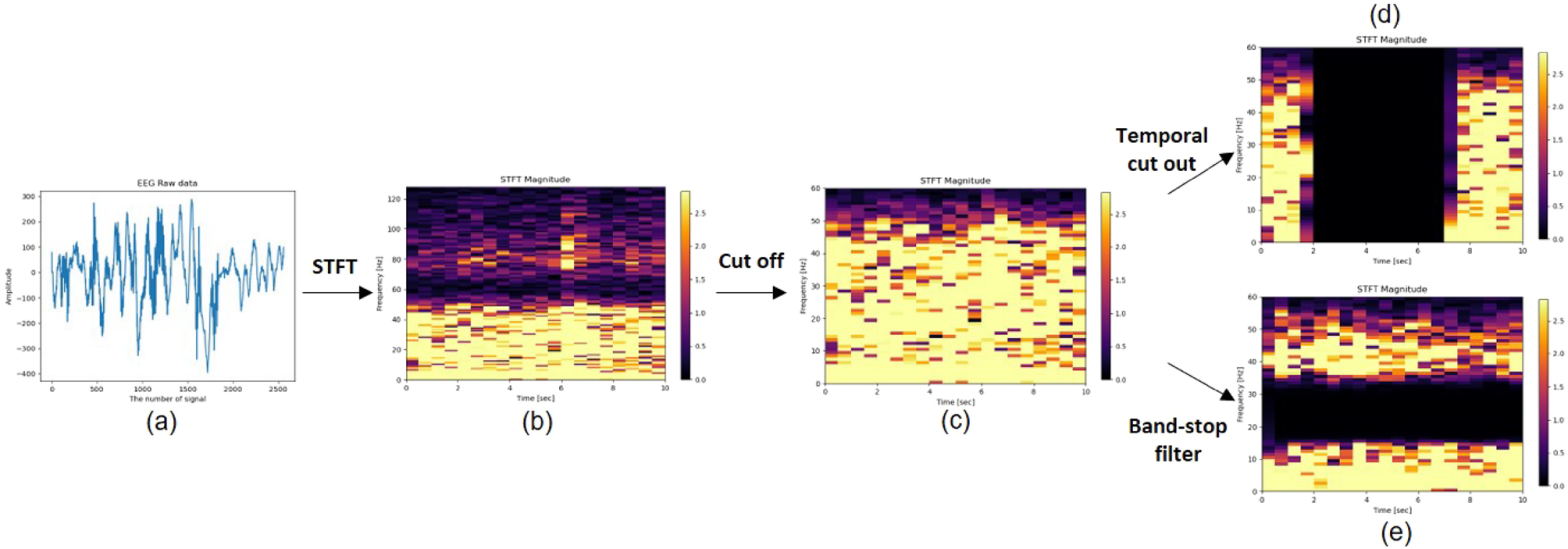
This is a pre-processing procedure for a single channel. The data shown in (**a**) is in its original form, referred to as raw EEG data. The spectrogram image depicted in (**b**) has undergone the application of STFT. The image (**c**) represents data that has been truncated to a frequency range of 0–60 Hz. For the pre-training phase, data augmentation techniques were applied to images (**d**, **e**). Specifically, temporal cut-out and band-stop filters were utilized.

The irregular and complex raw EEG data presented in Fig. 3a was transformed into a spectrogram image with the x-axis indicating time and the y-axis representing frequency using STFT, as shown in Fig. 3b. As a spectrogram, the power value of the frequency band at a particular time can be easily observed, and it can be analyzed using both the time information on the x-axis and the image characteristics.

1 $$\begin{aligned} STFT\{x(n)\}(m,\omega ) \equiv X(m,\omega ) = \Sigma _{n=-\infty } ^{\infty }x\left[ n\right] w[n-m]e^{-j\omega n} \end{aligned}$$

Equation (1) is converted into a discrete digital signal using STFT. Here, *x*[*n*] represents the raw signal in the time domain, m and n denote the time axes, and $\omega$ signifies the frequency axis. w[] refers to the window function. For continuous data analysis, the Hanning window function with a window length of 1 s and 50 $\%$ overlap was applied to enhance the time resolution^31^. As depicted in Fig. 3c, the data were constructed using only the information-rich data in the 0 $\sim$ 60 Hz band. Difficult-to-analyze EEG were transformed into spectrograms containing time-frequency information, and a preprocessing step was performed to facilitate feature extraction from the data.

## Pretext task: pre-training

We conducted pre-training to achieve high performance with a limited data. The original data were augmented using a band-stop filter and temporary cutout, and then trained within a model consisting of ResNet and supervised contrastive loss. Training with augmented data can prevent overfitting, and the image representation is acquired in advance. Even with a small dataset, the training model could determine optimal parameters through the use of the pre-trained ResNet.

### Data augmentation

The augmentation method has primarily been employed in image processing within the field of vision^32^, and it has also found applications in signal processing^33^ and other domains. For EEG data, which contains both signal information and STFT-converted image data, a band-stop filter and temporal cutout were employed to satisfy both requirements. The STFT-applied image takes the form of a horizontally and vertically cropped representation when specific frequency band and time zone information is removed. The images shown in Fig. 3d, e were generated through augmentation. The temporal cutout was vertically cropped, and 6 of 10 s were removed at random. The temporal cutout involved vertical cropping, removing 6 out of 10 seconds at random. Experiments were performed to determine the removed time and length of frequency information. Augmented images were used as input for the pre-trained model.

### Residual learning

CNN^34^, which is effective in analyzing patterns in images, has been widely utilized in the field of computer vision. Deeper layers within CNN models are recognized as crucial for determining the model’s performance. However, contrary to initial expectations, increasing the depth of layers in CNN-based models often leads to degradation issues^35^. ResNet was introduced as a solution to address this degradation problem. It employs the model structure of VGGNet (Visual Geometry Group Net)^36^ and incorporates shortcut connections to add input values to output values^35^.

**Figure 4:**
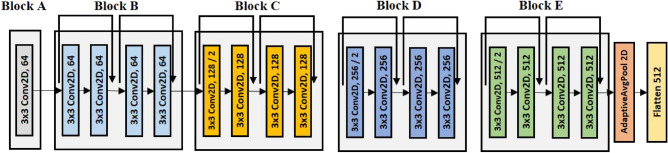
ResNet-18 architecture.

In this study, we employ ResNet-18, the shallowest model in the ResNet architecture. This decision is influenced by the experimental dataset, consisting of small images with dimensions of (21x60). Smaller images inherently carry less information, making it more challenging to effectively capture essential features and patterns within deeper networks. ResNet-18 consists of the five blocks, as illustrated in Fig. 4. Each block includes batch normalization, Rectified Linear Unit (ReLU), and max pooling. The input dimensions for CHB-MIT and SNUH datasets are (18 × 21 × 60) and (21 × 21 × 60), respectively. These dimensions represent the number of electrodes, the temporal information derived from a window size of 1 second and 50% overlap, and the frequency components.For CHB-MIT, the resulting feature maps from each block are as follows: (64 × 21 × 60), (64 × 21 × 60), (128 × 11 × 30), (256 × 6 × 15), and (512 × 3 × 8). The final feature map obtained from ResNet is transformed into a 512-dimensional vector through adaptive average pooling and a flatten layer. Throughout the pre-training and training processes, the output from ResNet is utilized as input values for the supervised contrastive loss and the LSTM layer, respectively.

### Supervised contrastive learning

Contrastive learning has its origins in metric learning^37^ and is currently primarily studied as a learning technique for pre-trained models. Among the notable approaches are self-supervised contrastive learning^38^ and supervised contrastive learning^39^. Self-supervised contrastive learning is an unsupervised learning algorithm that is appropriate for large quantities of unlabeled data, but it cannot outperform supervised learning. The proposed method to address these deficiencies is supervised contrastive learning. In contrast to self-supervised contrastive learning, loss values are allocated based on class. In other words, it is a method of supervised learning using labeled data. Equation (2) represents self-supervised contrastive loss, while Equation (3) denotes supervised contrastive learning.

2 $$\begin{aligned} L^{self} = - \Sigma _{i\in I} log{\exp (z_i \cdot z_j(i)/ \tau ) \over \Sigma _{ a \in A(i)} \exp (z_i \cdot z_a / \tau )} \end{aligned}$$

The symbol $\cdot$ represents the dot product, and $\tau$ is the hyperparameter. When the batch size is *N* and I $\equiv \{1\ldots 2N\}$ is the index of an augmented sample, 2*N* indexes are included. $z_j(i)$ represents a single positive sample, which is the remaining augmented image, while $2(N-1)$ indexes represent negative samples, denoted by $z_a$. In the denominator, equation $z_i$ $\cdot$ $z_a$ represents similarity comparisons for negative samples, and it is repeated $2(N-1)$ times. Only one image augmented from the same image has its numerator $z_i \cdot z_j(i)$ compared for similarity. With the exception of one augmented image, all images are considered negative.

3 $$\begin{aligned} L^{sup} = - \Sigma _{i\in I} {1\over |p(i)|} \Sigma _{p\in P(i)} log{\exp (z_i \cdot z_p/ \tau ) \over \Sigma _{ a \in A(i)} \exp (z_i \cdot z_a / \tau )} \end{aligned}$$

**Figure 5:**
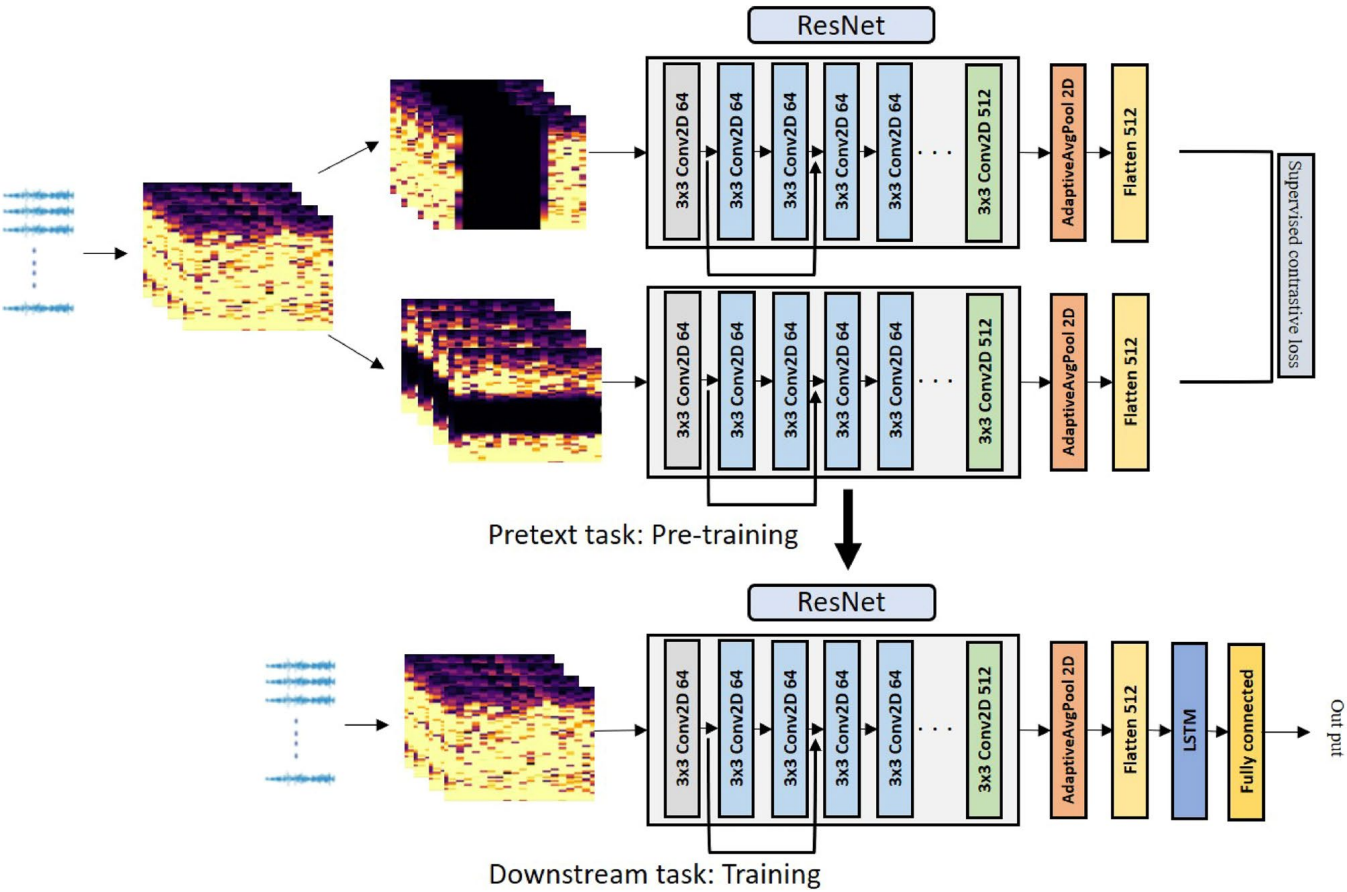
Our proposed method comprises two key modules: the Pretext task and the Downstream task. In the Pretext task, the data augmentation technique involving a band-stop filter and temporary cutout is applied, followed by the training of a pre-trained ResNet model with a supervised contrastive loss. This results in the generation of a pre-trained representation for the augmented data. In the Downstream task, fine-tuning is performed on the LSTM using the pre-trained ResNet, and training is conducted on the preprocessed original data.

Equation (3) *P*(*i*) represents a sample from the same class considered as positive. Positive and negative samples are separated by class, and the loss is calculated as the mean similarity value for all positive samples^39^. In supervised contrastive loss during training, the loss is determined by comparing data within the same batch. Therefore, the larger the positive sample size and batch size, the better the performance. We conducted pre-training using supervised contrastive learning, which clearly demonstrates distinctions between objects (Fig. 5).

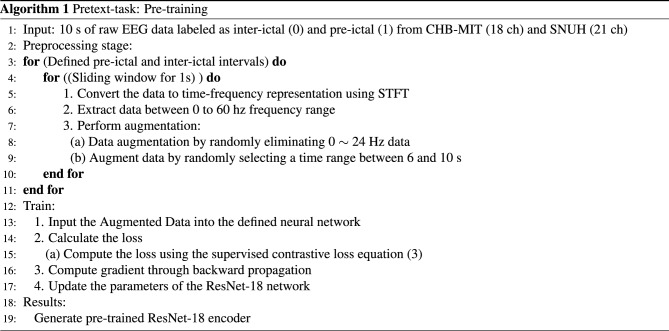

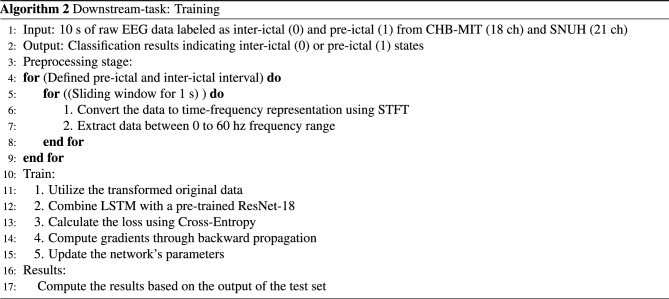





## Downstream task: training

### LSTM

**Figure 6:**
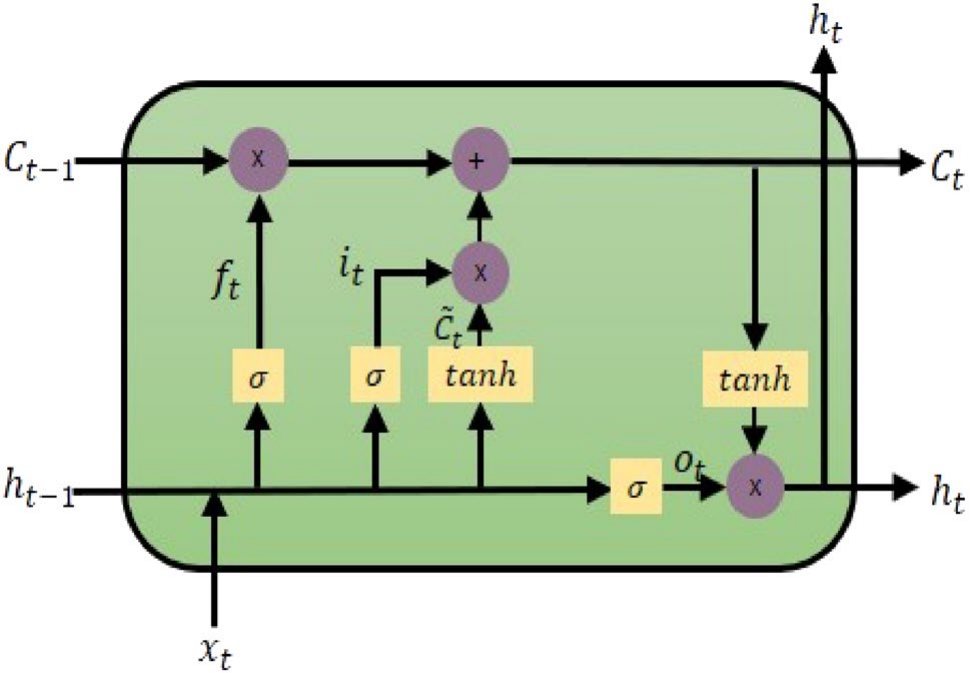
Structure of the LSTM cell.

As a deep learning model derived from the Recurrent Neural Network (RNN), the LSTM model has proven effective in multiple fields with time-dependent or sequence-based data, including speech recognition, language modeling, and translation. Additionally, to address the gradient vanishing phenomenon that occurs on long-term dependency data of RNN, it is possible to transmit information over long distances without losing it through the cell state. Figure 6 illustrates the internal structure of the LSTM cell state, encompassing the forget gate, input gate, and output gate.

- The LSTM’s calculation procedure is as follows: $C_t$ represents the cell state value, $h_t$ denotes the hidden state value, $x_t$ is the input value, $\sigma$ signifies the sigmoid function, *tanh* is the Hyperbolic Tangent function, and $f_t$, $i_t$, ${\tilde{C}}_t$ and $o_t$ represent the output values of each gate.
  - Equation (4) represents a forget gate. The sigmoid function produces a value ranging between 0 and 1, indicating the extent to which past information should be discarded. A value closer to 0 implies less retention of information.
    4 $$\begin{aligned} f_t=\sigma (W_f \cdot [h_{t-1}, x_t] + b_f) \end{aligned}$$
  - Equations (5) and (6) correspond to the input gate, responsible for selecting crucial information from incoming data. Equation (5) defines the value to be updated using the sigmoid function, while equation (6) calculates a new candidate value vector ${\tilde{C}}_t$, which will contribute to updating the cell state.
    5 $$\begin{aligned} i_t= & {} \sigma (W_i \cdot [h_{t-1}, x_t] + b) \end{aligned}$$
    6 $$\begin{aligned} {\tilde{C}}_t= & {} tanh(W_c\cdot [h_{t-1},x_t + b_c]) \end{aligned}$$
  - Equation (7) updates $C_{(t-1)}$ to $C_t$. This process involves updating the new cell state through a combination of addition and multiplication involving the data from the preceding steps. Specifically, Ct is updated by multiplying the previous cell state $C_{(t-1)}$ with the output ft from the forget gate, and it is further updated through a combination of multiplication and addition involving the values from the input gate.
    7 $$\begin{aligned} C_t = f_t*C_{t-1} + i_t * {\tilde{C}}_t \end{aligned}$$
  - Equations (8) and (9) represent an output gate responsible for generating the final output. In Equation (8), the sigmoid function determines the value of $x_t$ to be output. Ultimately, in Equation (9), the output is determined by multiplying the result obtained from Equation (8) with $C_t$.
  8 $$\begin{aligned} o_t= & {} \sigma (W_o[h_t-1,x_t]+b_o) \end{aligned}$$
  9 $$\begin{aligned} h_t= & {} \sigma _t*tanh(C_t) \end{aligned}$$

As demonstrated in the previous equation, the cell state selectively discards irrelevant past information, incorporates pertinent current information, and iteratively updates itself using the gates. This enables the LSTM model to exhibit outstanding performance, even when dealing with data that exhibits long-term dependencies^40^.

### ResNet-LSTM hybrid model

The STFT pre-processing step produces image data with time information along the x-axis and frequency information along the y-axis. In this study, time and frequency information was used to extract data characteristics using a hybrid model combining ResNet and LSTM. ResNet was used to extract the image features, which were extracted as 512-dimensional vector values. It was delivered to the LSTM as an input. Time-series analysis was performed on the extracted features using LSTM with one hidden layer. It was classified using a linear classifier with the dropout and ReLU layers in the output layer.

## Result and discussion

EEG data has three disadvantages in seizure prediction: complexity and irregularity, a small number of datasets, and imbalance. Patient-specific seizure prediction is more restricted by the separation of patient-specific data. Therefore, we developed a pre-trained model that can be applied to the prediction of seizures. To address the potential issue of overfitting due to limited training data, we employed the model described in the Pretext task process, as depicted in Fig. 5. This approach helped us reduce the risk of overfitting and improve the generalization of our model. Moreover, it provided initial weight values to determine the optimal training model parameters. The proposed method’s pseudocode is shown in Algorithm 1 and 2.

We defined a single data as 10 s and predicted seizures by classifying pre-ictal and inter-ictal data. Leave-one-out cross-validation was employed to aggregate the results effectively. In this approach, a pair of pre-ictal and inter-ictal data instances were treated as a singular unit, with N-1 units used for training while the remaining unit served as the testing set. This process was iterated N times. Evaluation metrics such as sensitivity, specificity, accuracy, and False Positive Rate (FPR) were employed and are detailed in Table 1. Furthermore, we conducted statistical testing on the means of each patient’s performance using a paired t-test. The training process utilized the window-based PyTorch framework and the Stochastic Gradient Descent (SGD) optimizer, which demonstrated superior performance in terms of generalization compared to adaptive optimization methods^41^. This offered an advantage in addressing overfitting concerns when dealing with limited data. For the pre-training phase, a batch size of 512, an epoch of 300, and a learning rate of 0.05 were employed. During the subsequent training phase, an epoch of 100, a learning rate of 0.01, and the same batch size were utilized. Each hyperparameter was determined through a series of experiments.

**Table 1:** Evaluation metrics.

Evaluation metrics	Calculation formula
Accuracy	(TP + TN)/(TP + TN + FP + FN)
Sensitivity	TP/(TP + FN)
Specificity	TN/(TN + FP)
FPR	FP/(FP + TN)

**Table 2:** Seizure prediction results obtained with the CHB-MIT dataset.

Patient	Pre-ictal: 10 min				Pre-ictal: 15 min				Pre-ictal: 30 min			
	Sensitivity (%)	Specificity (%)	Accuracy (%)	FPR	Sensitivity (%)	Specificity (%)	Accuracy (%)	FPR	Sensitivity (%)	Specificity (%)	Accuracy (%)	FPR
ResNet-LSTM												
chb01	99.58	99.69	99.63	0.003	98.58	98.71	98.64	0.013	88.27	93.30	90.79	0.067
chb02	79.36	86.63	82.99	0.134	72.11	85.13	78.62	0.149	61.03	95.70	78.36	0.043
chb03	83.76	96.82	90.29	0.032	86.84	99.61	93.22	0.004	91.61	99.33	95.47	0.007
chb04	69.33	92.64	80.99	0.074	79.57	92.90	86.24	0.071	66.75	94.42	80.58	0.056
chb05	72.67	90.99	81.83	0.090	79.29	91.78	85.54	0.082	79.36	91.70	85.53	0.083
chb06	93.25	95.98	94.62	0.040	91.12	98.29	94.70	0.017	87.48	95.72	91.60	0.043
chb07	94.75	96.90	95.83	0.031	96.89	96.52	96.71	0.035	97.52	97.97	97.75	0.020
chb08	99.36	98.68	99.02	0.013	99.37	99.12	99.25	0.009	90.91	79.40	85.15	0.206
chb09	50.04	87.35	68.70	0.126	51.52	88.97	70.24	0.110	56.11	87.07	71.59	0.129
chb10	81.97	89.12	85.55	0.109	80.87	88.18	84.53	0.118	78.50	78.91	78.71	0.211
chb11	100.00	99.66	99.83	0.003	99.89	95.23	97.56	0.048	N/A	N/A	N/A	N/A
chb12	94.97	97.00	95.98	0.030	87.49	92.88	90.19	0.071	71.80	93.38	82.59	0.066
chb13	73.85	90.45	82.15	0.095	77.24	85.90	81.57	0.141	75.30	89.07	82.18	0.109
chb14	79.55	91.60	85.58	0.084	79.52	88.27	83.89	0.117	64.67	73.28	68.97	0.267
chb15	68.20	61.39	64.80	0.386	52.06	86.49	69.28	0.135	52.18	66.14	59.16	0.339
chb16	78.82	82.64	80.73	0.174	74.10	90.91	82.51	0.091	7.43	89.67	48.55	0.103
chb17	97.63	98.20	97.91	0.018	97.61	98.99	98.30	0.010	97.34	98.32	97.83	0.017
chb18	71.49	97.76	84.62	0.022	74.89	97.03	85.96	0.030	73.99	95.53	84.76	0.045
chb19	86.97	99.83	93.40	0.002	96.35	98.88	97.62	0.011	96.65	99.66	98.16	0.003
chb20	98.22	98.31	98.27	0.017	98.95	98.90	98.92	0.011	92.02	99.25	95.63	0.008
chb21	78.09	83.21	80.65	0.168	82.94	78.73	80.84	0.213	77.96	47.01	62.49	0.530
chb22	82.06	94.02	88.04	0.060	87.21	100.00	93.60	0.000	91.35	99.39	95.37	0.006
chb23	99.72	93.23	96.47	0.068	99.78	96.63	98.20	0.034	98.86	97.89	98.37	0.021
chb24	82.39	80.45	81.42	0.196	83.97	84.03	84.00	0.160	75.12	78.98	77.05	0.210
Average	84.00	91.77	87.89	0.082	84.51	93.00	88.75	0.070	77.05	88.74	82.90	0.113

We include experimental results from all 24 patients. The experiment utilized pre-ictal intervals of 10, 15, and 30 min. The experiment utilized pre-ictal intervals of 10, 15, and 30 min. The corresponding table represents the outcome of without pre-training from ResNet-LSTM. Patient 11’s 30 min result was excluded from the analysis due to insufficient data.

**Table 3:** Seizure prediction results obtained with the CHB-MIT dataset.

	Pre-ictal: 10 min				Pre-ictal: 15 min				Pre-ictal: 30 min			
Patient	Sensitivity (%)	Specificity (%)	Accuracy (%)	FPR	Sensitivity (%)	Specificity (%)	Accuracy (%)	FPR	Sensitivity (%)	Specificity (%)	Accuracy (%)	FPR
Pre-train + ResNet-LSTM												
chb01	99.75	99.15	99.45	0.008	96.50	96.82	96.66	0.032	93.08	97.49	95.28	0.025
chb02	87.56	89.85	88.71	0.102	82.27	89.00	85.63	0.110	79.56	95.59	87.58	0.044
chb03	79.12	99.83	89.48	0.002	98.88	99.97	99.42	0.000	96.41	100.00	98.20	0.000
chb04	95.64	95.05	95.35	0.049	88.64	95.62	92.13	0.044	69.71	96.23	82.97	0.038
chb05	79.40	84.39	81.90	0.156	84.79	91.95	88.37	0.081	74.86	98.77	86.81	0.012
chb06	93.44	98.08	95.76	0.019	92.54	97.08	94.81	0.029	91.11	96.92	94.02	0.031
chb07	92.39	96.79	94.59	0.032	94.73	97.94	96.33	0.021	95.25	99.01	97.13	0.010
chb08	99.59	99.59	99.59	0.004	100.00	99.24	99.62	0.008	97.07	79.97	88.52	0.200
chb09	89.72	97.50	93.61	0.025	91.08	98.20	94.64	0.018	89.36	97.65	93.51	0.023
chb10	80.47	91.59	86.03	0.084	97.45	90.81	94.13	0.092	95.03	95.74	95.38	0.043
chb11	99.83	100.00	99.92	0.000	99.94	97.92	98.93	0.021	N/A	N/A	N/A	N/A
chb12	98.96	99.26	99.11	0.007	93.96	97.66	95.81	0.023	79.23	92.18	85.71	0.078
chb13	92.14	93.57	92.86	0.064	94.84	96.39	95.61	0.036	82.55	97.17	89.86	0.028
chb14	83.38	90.74	87.06	0.093	72.39	94.16	83.28	0.058	70.32	83.45	76.88	0.165
chb15	72.80	58.64	65.72	0.414	68.05	76.81	72.43	0.232	36.13	84.76	60.44	0.152
chb16	78.75	90.56	84.65	0.094	72.67	83.56	78.11	0.164	12.76	82.52	47.64	0.175
chb17	99.66	99.10	99.38	0.009	99.18	99.85	99.51	0.001	98.57	98.46	98.51	0.015
chb18	75.30	96.70	86.00	0.033	73.18	95.48	84.33	0.045	74.58	99.64	87.11	0.004
chb19	97.72	99.49	98.60	0.005	84.96	96.97	90.97	0.030	99.05	99.83	99.44	0.002
chb20	99.27	99.13	99.20	0.009	99.16	98.73	98.94	0.013	97.18	99.05	98.12	0.009
chb21	78.68	85.15	81.92	0.148	84.68	79.63	82.15	0.204	80.63	62.80	71.71	0.372
chb22	81.11	96.11	88.61	0.039	100.00	99.66	99.83	0.003	84.37	99.47	91.92	0.005
chb23	100.00	98.08	99.04	0.019	100.00	95.77	97.89	0.042	98.41	98.06	98.23	0.019
chb24	85.51	82.16	83.83	0.178	81.45	90.44	85.95	0.096	80.13	77.50	78.81	0.225
**Average**	**89.17** **(+5.17)**	**93.35** **(+1.58)**	**91.26** **(+3.37)**	**0.066** **(− 0.016)**	**89.64** **(+5.13)**	**94.15** **(+1.15)**	**91.90** **(+3.15)**	**0.058** **(− 0.012)**	**81.54** **(+4.49)**	**92.71** **(+3.97)**	**87.12** **(+4.22)**	**0.073** **(− 0.040)**

We include experimental results from all 24 patients. The experiment utilized pre-ictal intervals of 10, 15, and 30 min. The experiment utilized pre-ictal intervals of 10, 15, and 30 min. The corresponding table demonstrates the outcomes of deploying the pre-train model to ResNet-LSTM. Patient 11’s 30 min result was excluded from the analysis due to insufficient data. Significant values are in bold.

**Table 4:** Seizure prediction results obtained with the SNUH dataset.

	Pre-ictal: 10 min				Pre-ictal: 15 min				Pre-ictal: 30 min			
Patient	Sensitivity (%)	Specificity (%)	Accuracy (%)	FPR	Sensitivity (%)	Specificity (%)	Accuracy (%)	FPR	Sensitivity (%)	Specificity (%)	Accuracy (%)	FPR
ResNet-LSTM												
snuh01	55.67	77.33	66.50	0.227	66.78	72.33	69.56	0.277	62.84	32.47	47.65	0.675
snuh02	98.76	98.42	98.59	0.016	95.40	81.00	88.20	0.190	83.14	90.71	86.93	0.093
snuh03	70.20	89.63	79.91	0.104	70.32	82.09	76.21	0.179	77.42	86.03	81.72	0.140
snuh04	7.87	94.84	51.35	0.052	8.02	93.77	50.90	0.062	52.82	82.38	67.60	0.176
snuh05	70.52	47.93	59.22	0.521	81.34	58.95	70.15	0.410	61.47	66.89	64.18	0.331
snuh06	78.68	72.45	75.57	0.275	76.50	66.33	71.41	0.337	80.78	61.25	71.02	0.387
snuh07	99.07	92.72	95.90	0.073	98.60	99.49	99.05	0.005	92.38	98.58	95.48	0.014
snuh08	86.59	77.75	82.17	0.223	81.00	82.55	81.78	0.175	62.94	89.89	76.42	0.101
snuh09	81.95	53.69	67.82	0.463	46.20	70.45	58.32	0.296	75.56	67.37	71.47	0.326
snuh10	39.42	81.50	60.46	0.185	56.27	72.65	64.46	0.273	43.70	72.66	58.18	0.273
snuh11	21.88	49.13	35.50	0.509	25.07	38.53	31.80	0.615	24.40	45.11	34.76	0.549
snuh12	98.82	79.61	89.21	0.204	98.15	92.87	95.51	0.071	81.74	80.68	81.21	0.193
snuh13	21.83	97.38	59.60	0.026	67.79	86.14	76.96	0.139	96.40	67.17	81.78	0.328
snuh14	68.02	68.02	68.02	0.320	60.61	66.39	63.50	0.336	69.37	67.36	68.37	0.326
snuh15	54.60	84.04	69.32	0.160	49.83	81.41	65.62	0.186	69.10	73.81	71.46	0.262
snuh16	93.15	99.41	96.28	0.006	87.37	98.18	92.77	0.018	80.96	95.48	88.22	0.045
snuh17	72.25	96.33	84.29	0.037	69.66	95.96	82.81	0.040	82.30	95.81	89.06	0.042
snuh18	96.05	96.11	96.08	0.039	94.91	96.67	95.79	0.033	95.38	95.68	95.53	0.043
snuh19	64.21	94.42	79.31	0.056	66.89	94.67	80.78	0.053	42.38	95.78	69.08	0.042
snuh20	30.40	68.98	49.69	0.310	23.27	66.59	44.93	0.334	52.28	59.54	55.91	0.405
snuh21	90.44	95.71	93.08	0.043	84.81	93.70	89.25	0.063	76.69	91.35	84.02	0.087
snuh22	63.96	79.58	71.77	0.204	54.79	74.15	64.47	0.259	56.72	61.81	59.26	0.382
snuh23	82.66	93.32	87.99	0.067	73.06	94.16	83.61	0.058	87.91	95.78	91.85	0.042
snuh24	40.27	80.88	60.58	0.191	45.68	82.27	63.97	0.177	28.92	47.01	37.97	0.530
Average	66.14	82.05	74.09	0.180	65.93	80.89	73.41	0.191	68.23	75.86	72.05	0.241

We include experimental results from all 24 patients. The experiment utilized pre-ictal intervals of 10, 15, and 30 min. The experiment utilized pre-ictal intervals of 10, 15, and 30 min. The corresponding table represents the outcome of without pre-training from ResNet-LSTM.

**Table 5:** Seizure prediction results obtained with the SNUH dataset.

	Pre-ictal: 10 min				Pre-ictal: 15 min				Pre-ictal: 30 min			
Patient	Sensitivity (%)	Specificity (%)	Accuracy (%)	FPR	Sensitivity (%)	Specificity (%)	Accuracy (%)	FPR	Sensitivity (%)	Specificity (%)	Accuracy (%)	FPR
Pre-train + ResNet-LSTM												
snuh01	88.66	98.39	93.53	0.016	91.75	97.64	94.70	0.024	86.52	76.35	81.43	0.236
snuh02	99.10	100.00	99.55	0.000	88.18	99.63	93.90	0.004	86.99	99.94	93.47	0.001
snuh03	85.42	97.10	91.26	0.029	90.35	95.86	93.11	0.041	90.37	98.40	94.39	0.016
snuh04	99.83	9.81	54.82	0.902	99.10	11.84	55.47	0.882	98.99	35.34	67.17	0.647
snuh05	90.27	77.66	83.97	0.223	95.88	63.44	79.66	0.366	93.29	86.46	89.87	0.135
snuh06	83.99	89.68	86.84	0.103	88.15	89.88	89.01	0.101	90.51	73.05	81.78	0.269
snuh07	99.92	97.88	98.90	0.021	99.94	98.82	99.38	0.012	98.49	97.77	98.13	0.022
snuh08	86.46	80.88	83.67	0.191	79.24	83.39	81.31	0.166	71.00	88.24	79.62	0.118
snuh09	97.46	42.47	69.97	0.575	83.16	41.94	62.55	0.581	81.35	90.77	86.06	0.092
snuh10	41.29	87.48	64.38	0.125	61.17	80.73	70.95	0.193	59.45	89.65	74.55	0.103
snuh11	55.22	80.15	67.68	0.199	49.38	72.43	60.91	0.276	41.11	69.31	55.21	0.307
snuh12	100.00	100.00	100.00	0.000	99.49	100.00	99.75	0.000	87.13	81.27	84.20	0.187
snuh13	54.57	100.00	77.28	0.000	99.05	100.00	99.52	0.000	100.00	96.23	98.12	0.038
snuh14	92.30	82.66	87.48	0.173	85.02	85.91	85.47	0.141	73.06	91.12	82.09	0.089
snuh15	81.56	90.86	86.21	0.091	90.72	96.00	93.36	0.040	66.76	93.17	79.96	0.068
snuh16	91.24	99.53	95.39	0.005	84.93	99.02	91.98	0.010	92.03	98.23	95.13	0.018
snuh17	85.90	98.03	91.96	0.020	90.39	99.63	95.01	0.004	93.90	98.77	96.33	0.012
snuh18	94.87	97.97	96.42	0.020	93.98	98.95	96.46	0.010	96.18	98.62	97.40	0.014
snuh19	99.58	98.82	99.20	0.012	71.38	98.48	84.93	0.015	86.85	98.94	92.90	0.011
snuh20	49.86	84.21	67.03	0.158	48.48	77.52	63.00	0.225	64.30	73.89	69.10	0.261
snuh21	94.30	99.69	97.00	0.003	89.75	97.66	93.71	0.023	84.22	97.87	91.04	0.021
snuh22	51.95	78.79	65.37	0.212	50.24	79.09	64.67	0.209	58.80	78.67	68.74	0.213
snuh23	31.73	98.31	65.02	0.017	43.94	98.20	71.07	0.018	65.94	98.32	82.13	0.017
snuh24	61.93	94.16	78.05	0.058	46.86	81.54	64.20	0.185	10.52	85.46	47.99	0.145
**Average**	**79.89** **(+13.75)**	**86.86** **(+4.81)**	**83.37** **(+9.28)**	**0.131** **(− 0.049)**	**80.02** **(+14.09)**	**85.32** **(+4.43)**	**82.67** **(+9.26)**	**0.147** **(− 0.044)**	**78.24** **(+10.01)**	**87.33** **(+11.47)**	**82.78** **(+10.73)**	**0.127** **(− 0.114)**

We include experimental results from all 24 patients. The experiment utilized pre-ictal intervals of 10, 15, and 30 min. The experiment utilized pre-ictal intervals of 10, 15, and 30 min. The corresponding table demonstrates the outcomes of deploying the pre-train model to ResNet-LSTM. Significant values are in bold.

**Table 6:** The corresponding table presents a comparison and summary of the experimental results obtained using pre-trained models on the CHB-MIT dataset.

	Sensitivity (%)	Specificity (%)	Accuracy (%)	FPR	Sensitivity (%)	Specificity (%)	Accuracy (%)	FPR	*p*-value
10 min	84.00	91.77	87.89	0.082	**89.17** **(+5.17)**	**93.35** **(+1.58)**	**91.26** **(+3.37)**	**0.066** **(− 0.016)**	**0.009**
15 min	84.51	93.00	88.75	0.070	**89.64** **(+5.13)**	**94.15** **(+1.15)**	**91.90** **(+3.15)**	**0.058** **(− 0.012)**	**0.024**
30 min	77.05	88.74	82.90	0.113	**81.54** **(+4.49)**	**92.71** **(+3.97)**	**87.12** **(+4.22)**	**0.073** **(− 0.040)**	**0.002**

The left and right sides of the table show the results before and after the pre-trained model application, respectively. The *p*-value represents the result of the paired t-test. Significant values are in bold.

**Table 7:** The corresponding table presents a comparison and summary of the experimental results obtained using pre-trained models on the SNUH dataset.

	Sensitivity (%)	Specificity (%)	Accuracy (%)	FPR	Sensitivity (%)	Specificity (%)	Accuracy (%)	FPR	*p*-value
10 min	66.14	82.05	74.09	0.180	**79.89** **(+13.75)**	**86.86** **(+4.81)**	**83.37** **(+9.28)**	**0.131** **(− 0.049)**	**0.044**
15 min	65.93	80.89	73.41	0.191	**80.02** **(+14.09)**	**85.32** **(+4.43)**	**82.67** **(+9.26)**	**0.147** **(− 0.044)**	**0.001**
30 min	68.23	75.86	72.05	0.241	**78.24** **(+10.01)**	**87.33** **(+11.47)**	**82.78** **(+10.73)**	**0.127** **(− 0.114)**	**0.000**

The left and right sides of the table show the results before and after the pre-trained model application, respectively. The *p*-value represents the result of the paired t-test. Significant values are in bold.

**Figure 7:**
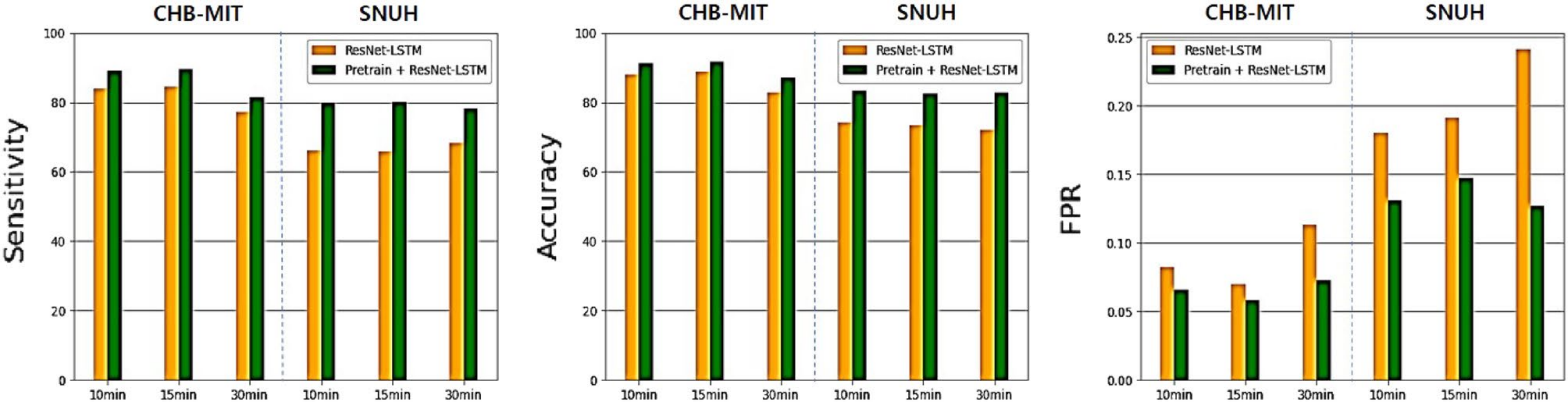
The graph provides the comparison of two models, ResNet-LSTM and Pre-train + ResNet-LSTM. The evaluation metrics comprised sensitivity, accuracy, and FPR.

In this paper, the SNUH and CHB-MIT datasets were utilized for validation. Both datasets share the same number of patients, as detailed in the “DATABASE” section. However, the SNUH dataset contains 78 fewer instances of ictals and is measured using the noisier unipolar reference method. In our experimental setup, we defined the pre-ictal period as 10, 15, and 30 min, with subsequent evaluation metrics confirming the outcomes for each respective period. Tables 2, 3, 4 and 5 present patient-specific the results. Tables 3 and 5 are the results of ResNet-LSTM without applying the pre-trained model, and Tables 3 and 5 are the results of applying the pre-train model. A summary of the results’ performance is provided in Tables 6 and 7. According to Table 6, all results using pre-train were enhanced, with sensitivity showing improvement relative to specificity in the 10 and 15 min data. In the case of the 30-min data, a higher rate of increase in specificity and FPR led to an improvement in accuracy. Similar to the results obtained from the CHB-MIT, all SNUH results in Table 7 also improved, and the sensitivity of the 10 and 15 min results improved even further. In addition, the specificity was enhanced in the 30 min data. Experiments conducted on both datasets yielded comparable outcomes. The 30 min pre-ictal period presented fewer extractable data compared to the 10 and 15-min periods, and seizure signs tended to weaken as time distanced from the ictal event. Consequently, when comparing the 30 min data to other time intervals, further enhancements in specificity were observed. In the context of seizure prediction, defining the pre-ictal period is a significant consideration. Extending the pre-ictal period offers the advantage of advanced patient preparation, but it comes with the trade-off of reduced accuracy and increased patient anxiety. As demonstrated in Fig. 7, using the two datasets, CHB-MIT had the highest value at 15 min, while SNUH had the highest value at 10 min, and both datasets had similar values at 10 and 15 min. Even with a small amount of data, accuracy for 10, 15 min was ensured in SNUH, and in the paired t-test results of Tables 6 and 7, the numerical values according to the presence or absence of pre-training showed a significant difference in the overall result (p<0.05), indicating that pre-training plays a significant role in improving the numerical value.

**Table 8:** The following table displays the hybrid model’s performance verification results.

Dataset	Method	Pre-train + ResNet				Pre-train + ResNet - LSTM			
		Sensitivity (%)	Specificity (%)	Accuracy (%)	FPR	Sensitivity (%)	Specificity (%)	Accuracy (%)	FPR
	10 min	88.94	92.33	90.64	0.076	**89.17** **(+0.23)**	**93.35** **(+1.02)**	**91.26** **(+0.62)**	**0.066** **(− 0.010)**
CHB-MIT	15min	89.14	93.92	91.53	0.06	**89.64** **(+0.50)**	**94.15** **(+0.23)**	**91.90** **(+0.37)**	**0.058** **(− 0.020)**
	30 min	81.08	91.67	86.38	0.083	**81.54** **(+0.46)**	**92.71** **(+1.04)**	**87.12** **(+0.74)**	**0.073** **(− 0.010)**
	10 min	73.84	85.43	79.64	0.146	**79.89** **(+6.05)**	**86.86** **(+1.43)**	**83.37** **(+3.73)**	**0.131** **(− 0.015)**
SNUH	15 min	75.05	85.22	80.14	0.148	**80.02** **(+4.97)**	**85.32** **(+0.10)**	**82.67** **(+2.53)**	**0.147** **(− 0.001)**
	30 min	75.53	84.01	79.77	0.16	**78.24** **(+2.71)**	**87.33** **(+3.32)**	**82.78** **(+3.01)**	**0.127** **(− 0.033)**

The left side of the table presents outcomes for the pre-trained ResNet, the right side displays the results for the hybrid model that integrates both pre-trained ResNet and LSTM. The table is organized with the outcomes for the CHB-MIT dataset in the upper section and the SNUH dataset in the lower section. Significant values are in bold.

STFT conversion transforms the EEG data into a spectrogram image with represented on the x-axis and frequency on the y-axis. For training, we used a hybrid model that combines ResNet and LSTM to reflect both types of information. The experimental outcomes for pre-train + ResNet and pre-train + ResNet-LSTM are outlined in Table 8. As a result of the experiment, improved results were obtained for both datasets, confirming the benefits of the hybrid model.

**Table 9:** This table presents the outcomes of conventional seizure prediction methods on the CHB-MIT dataset.

Authors	Year	Dataset	Number of patients	Classifier	Features	Acc (%)	Sen (%)	Spec (%)	FPR
Khan *et al.*^43^	2017	CHB-MIT	**15 patients**	CNN	Wavelet Transform	N/A	87.8	N/A	0.142
Truong *et al.*^44^	2018	CHB-MIT	**13 patients**	CNN	Short time Fourier Transform	N/A	81.2	N/A	0.160
Ozcan *et al.*^25^	2019	CHB-MIT	**16 patients**	3D-CNN	Spectral band power, statistical moment, Hjorth parameters	N/A	85.7	N/A	0.096
Romney *et al.*^45^	2020	CHB-MIT,	**23 patients**	MAML	Ensemble Empirical Mode Decomposition	N/A	86.7	89.5	N/A
Yang*et al.*^42^	2021	CHB-MIT	**13 patients**	RDANet	Short time Fourier Transform	92.1	89.3	92.7	N/A
Jemal *et al.*^46^	2022	CHB-MIT	**23 patients**	CNN	Raw data	90.9	96.1	84.7	0.040
**This work 1**	2022	CHB-MIT	**13 patients**	**Pre-train + ResNet-LSTM**	Short time Fourier Transform	**92.3**	**90.0**	**94.5**	**0.055**
**This work 2**	2022	CHB-MIT	**24 patients**	**Pre-train + ResNet-LSTM**	Short time Fourier Transform	**91.9**	**89.6**	**94.2**	**0.059**

“This work 1” corresponds to the validation results for the same patient as in the prior method^42^. “This work 2” signifies the pre-ictal 15 min overall outcomes encompassing all patients. Significant values are in bold.

Table 9 shows a previous study conducted on patient-specific seizure prediction using the CHB-MIT dataset. Contemporary research trends involve extracting data in the frequency domain as features and utilizing machine learning and deep learning methodologies as classifiers. Ongoing investigations aim to enhance sensitivity and reduce FPR by addressing challenges such as data imbalance and insufficient samples, both inherent in EEG. Jemal *et al.*^46^ obtained a high sensitivity of 96.1% from 23 patients but with low specificity, and they employed 5-fold cross-validation instead of Leave-one-out cross-validation as the performance validation method. Table 9 includes two approaches^42,44^ employ STFT, the same method applied in this study. Among these, Yang *et al.*^42^ experimental results demonstrated low sensitivity of 59.9%, 66%, and 56% for patients 2,9, and 14, respectively. For patients 2 and 9, limited pre-ictal data relative to the total duration was a factor, while patient 14 had a shorter recording duration, indicating less effective training. The majority of studies on seizure prediction using CHB-MIT reported poor patient outcomes due to the aforementioned issues. As demonstrated in Table 3, the experimental results of our study revealed that the 10 min sensitivity for all three patients exceeded 80%, and patient 9’s sensitivity improved by nearly 40%. The inter-ictal weight concentration phenomenon was resolved by addressing the class imbalance. By generating a pre-trained model, the representation was acquired in advance, enabling the model to determine the optimal weight values during the actual training process. Through these interventions, we succeeded in enhancing outcomes for patients with previously low sensitivity. Table 9 does not present results based on all 24 patients, as certain experimental patient data was lacking and there was no common channel. The proposed method’s experimental results were presented for all patients, including those used in the previous method^42^. We obtained higher sensitivity and lower FPR compared to conventional methods.

## Conclusion

In this paper, we propose a method for predicting epilepsy seizures based on a pre-trained model that employs supervised contrastive learning and a hybrid model that combines ResNet and LSTM. In the pre-processing phase, the data were transformed using STFT to ensure that the training model could efficiently perform feature analysis, and the class imbalance between pre-ictal and inter-ictal as well as the insufficient data were addressed by sampling and oversampling. During pre-training, data were augmented and pre-trained with a ResNet and supervised contrastive loss model so that the training model could find the optimal parameter with fewer data. During the training phase, image features and time series data were extracted using a hybrid model comprised a pre-trained ResNet and LSTM. The experimental results reveal that CHB-MIT demonstrates optimal performance for the 15 min pre-ictal period, while SNUH performs best for the 10 min pre-ictal period. We demonstrated greater sensitivity and a lower FPR than conventional methods.

## Data Availability

The CHB-MIT data used in this study are public database, which could be accessed and downloaded from https://archive.physionet.org/physiobank/database/chbmit/. The SNUH data used in this study are not publicly available. The data may be made available from the corresponding authors upon reasonable request subject to permission and approval from the corresponding organizations and institutional review boards.
